# Mechanism for oral tumor cell lysyl oxidase like-2 in cancer development: synergy with PDGF-AB

**DOI:** 10.1038/s41389-019-0144-0

**Published:** 2019-05-13

**Authors:** Faranak Mahjour, Vrinda Dambal, Neha Shrestha, Varun Singh, Vikki Noonan, Alpdogan Kantarci, Philip C. Trackman

**Affiliations:** 1 Boston University Henry M. Goldman School of Dental Medicine, Department of Molecular and Cell Biology, Boston, MA 02118 USA; 2 Boston University Henry M. Goldman School of Dental Medicine, Division of Oral & Maxillofacial Pathology, Boston, MA 02118, USA; 3 Forsyth Institute, Cambridge, MA 02142 USA

**Keywords:** Cancer, Cell biology

## Abstract

Extracellular lysyl oxidases (LOX and LOXL1–LOXL4) are critical for collagen biosynthesis. LOXL2 is a marker of poor survival in oral squamous cell cancer. We investigated mechanisms by which tumor cell secreted LOXL2 targets proximal mesenchymal cells to enhance tumor growth and metastasis. This study identified the first molecular mechanism for LOXL2 in the promotion of cancer via its enzymatic modification of a non-collagenous substrate in the context of paracrine signaling between tumor cells and resident fibroblasts. The role and mechanism of active LOXL2 in promoting oral cancer was evaluated and employed a novel LOXL2 small molecule inhibitor, PSX-S1C, administered to immunodeficient, and syngeneic immunocompetent orthotopic oral cancer mouse models. Tumor growth, histopathology, and metastases were monitored. In vitro mechanistic studies with conditioned tumor cell medium treatment of normal human oral fibroblasts were carried out in the presence and absence of the LOXL2 inhibitor to identify signaling mechanisms promoted by LOXL2 activity. Inhibition of LOXL2 attenuated cancer growth and lymph node metastases in the orthotopic tongue mouse models. Immunohistochemistry data indicated that LOXL2 expression in and around tumors was decreased in mice treated with the inhibitor. Inhibition of LOXL2 activity by administration of PXS-S1C to mice reduced tumor cell proliferation, accompanied by changes in morphology and in the expression of epithelial to mesenchymal transition markers. In vitro studies identified PDGFRβ as a direct substrate for LOXL2, and indicated that LOXL2 and PDGF-AB together secreted by tumor cells optimally activated PDGFRβ in fibroblasts to promote proliferation and the tendency toward fibrosis via ERK activation, but not AKT. Optimal fibroblast proliferation in vitro required LOXL2 activity, while tumor cell proliferation did not. Thus, tumor cell-derived LOXL2 in the microenvironment directly targets neighboring resident cells to promote a permissive local niche, in addition to its known role in collagen maturation.

## Introduction

Oral squamous cell carcinoma (OSCC) accounts for more than 90% of oral cavity cancers and is the sixth most common cancer in the world^1,2^. Tobacco smoking and excessive alcohol are major risk factors with synergistic effects^3^. Metastasis to cervical lymph nodes in patients with OSCC occurs in almost half of patients^4^ and recurrent metastasis occurs in 20–30% of patients after treatment^5,6^. These cancers have a poor 5-year survival rate, and cause significant morbidity due to limiting speech, food intake, and other aspects of oral and systemic health.

Lysyl oxidases (LOXs) catalyze the oxidative deamination of the ɛ-amino group of lysine and hydroxylysine residues in the telopeptide regions of procollagens to form peptidyl aldehydes, which results in biosynthetic cross-linking of collagen^7^. Overexpressed LOXs promote cancer progression in part due to excess modification of the extracellular matrix that can stimulate invasion and metastasis^8^. The LOX family consists of five members: *LOX* and the four related genes *LOX*-like-1–4 (*LOXL1–LOXL4*). Cancer progression and metastasis depends on the interactions between cancer cells and the tumor microenvironment, modifying tumor and stromal cell proliferation, extracellular matrix production and turnover, drug resistance, cell migration, and metastasis. Collagen accumulation, fibrosis, and dense or stiff microenvironments promote metastasis in solid tumors including breast cancer through focal adhesion formation^8,9^. LOXs may additionally interact with and/or oxidize other non-collagen proteins, which in turn regulate cell signaling pathways^10–13^, which can modulate cancer progression.

LOX-like-2 (LOXL2) has emerged as a biomarker for OSCC and its overexpression is associated with poor prognosis in patients^14^. The underlying mechanisms of the effect of LOXL2 on invasiveness of cancer are not well understood. LOXL2 nuclear interaction with Snail that regulates E-cadherin and leads to epithelial–mesenchymal transition (EMT) and invasiveness has been suggested^13^. Other studies suggested that interaction of LOXL2 with histones and nuclear proteins leads to EMT and invasiveness. However, there is no evidence of direct oxidation of these nuclear proteins by LOXL2 and the mechanism of action of LOXL2 remains unclear^7,13,15^. With a poor survival rate of OSCC, high occurrence of metastasis, and difficulties faced by patients with conventional treatments, there is a clear need to develop new targeted therapeutic approaches to address oral cancer and metastasis. The current study determined the effects and mechanism of secreted LOXL2 as a mediator of the progression and invasiveness of OSCC in two orthotopic in vivo mouse models, employing a novel LOXL2 pharmacologic small molecule inhibitor. Reduced tumor growth and metastasis and apparent inhibition of EMT was found in response to the inhibitor. Studies of mechanisms in vitro support that tumor cell-derived LOXL2 directly stimulated stromal fibroblast proliferation and activity by enhancing PDGF-AB-mediated signaling after modification of PDGFRβ, while having no proliferative effect on tumor cells themselves. These interactions define a novel interaction that occurs in the microenvironment emanating from tumor cells and that targets stromal mesenchymal cells. These findings provide a mechanistic basis for potential novel therapeutic approaches to address tumor growth, fibrosis, and metastasis.

## Methods

### Histopathology of LOXL2 and LOX expression in human oral cancer biopsies

Tissue blocks from three to five biopsies from different donors corresponding to dysplasia, differentiated OSCC, and poorly differentiated oral cancer, respectively, from the Boston University School of Dental Medicine Pathology diagnostic services laboratory were obtained under an approved IRB protocol (#31869). Sections (6 µm) were stained with hematoxylin and eosin, or immunostained for LOX or LOXL2 with counterstaining with hematoxylin^16,17^.

### Orthotopic tongue immunodeficient mouse model

All animal experiments were approved by the Boston University Institutional Animal Care and Use Committee, protocol #15354. HSC3 cells were a generous gift from Dr. Roberto Weigert (NIDCR, Bethesda, MD) and were validated by STR profiling by Genetica DNA Laboratories at the time of initiation of the studies. Cells were mycoplasma-free prior to use. HSC3 cells transduced with a lentivirus expressing the red fluorescent protein (RFP) DsRed were cultured according to the methods described previously^18^. Cell suspension (480,000 cells in 40 µl) was injected to the tongue of each nude mouse (NCRNU mice 6-week-old female, Taconic) under anesthesia (3% isoflurane). Diet gels containing electrolytes and nutrients were made available to the mice. Tumor growth was measured by caliper measurements and distant metastasis was detected by IVIS in mice injected with HSC3 ectopically expressing DsRed. There were four experimental groups (*n* = 8 per group) in this in vivo experiment: 1. Mice injected with HSC3 cells transduced with DsRed; 2. Mice injected with HSC3 cells transduced with DsRed and the LOXL2 inhibitor PXS-S1C (30 mg/kg in DMSO i.p.; three times per week); 3. Mice injected with HSC3 cells transduced with DsRed, PXS-S1C (10 mg/kg in DMSO i.p.; three times per week); 4. Control, group mice with no HSC3 injected cells, and no PXS-1C applied to serve as IVIS negative controls. Three weeks later, the mice were euthanized using isoflurane overdose followed by cervical dislocation.

### Orthotopic tongue immunocompetent mouse model

LY2 cells were cultured and processed as above. Aliquots of 40 µl of the cell suspension (480,000 cells) were injected to the tongue of each immunocompetent mouse (6-week-old female BALB/c) under anesthesia (3% isoflurane), and diet gels containing electrolytes and nutrients (76A, ClearH20) were made available to the mice. There were four experimental groups in this in vivo experiment (*n* = 12 per group): 1. Mice injected with LY2 cells; 2. Mice injected with LY2 cells, PXS-S1C (30 mg/kg in DMSO i.p., three times per week); 3. Mice injected with LY2 cells, PXS-S1C (10 mg/kg in DMSO i.p., three times per week); 4. Control, group mice (*n* = 8, without injection). Mice were euthanized after 6 weeks.

### LOXL2 inhibitor treatment of mice

PXS-S1C was first dissolved in sterile DMSO at 2.2 or 6.6 mg/ml for a dosing of 10 or 30 mg/kg, respectively. PSX-S1C was administered by intraperitoneal injection 12–24 h before the injection of cancer cells. The PXS-S1C injection was continued at a frequency of three times per week.

The tongues and lymph nodes were fixed in 4% paraformaldehyde, paraffin-embedded and were sectioned (6 µm). Tongues were fixed for histology, hematoxylin and eosin staining, Sirius red staining (Sirius red in picric acid), and immunohistochemistry (IHC) staining with anti-Ki67 antibody (ab15580, Abcam) or anti-proliferating cell nuclear antigen antibody (PCNA, ab92552, Abcam), anti-LOXL2 antibody (GTX105085, GeneTex), anti-E-cadherin antibody (610181, BD Transduction Laboratories), and anti-vimentin antibody (ab92547, Abcam). The images of the stained slides were taken with a Zeiss microscope with lens objectives of ×4, ×10, and ×20. Analyses of IHC slides was performed in a blinded fashion by a lab member, who was not informed regarding the identity of each experimental group.

### Cell culture

Human gingival fibroblasts (HGF) and cancer cells were grown separately at 37 °C and 5% CO_2_ in a humidified incubator. The HGF were taken from three healthy donors aged 25–35 years undergoing crown lengthening procedures. The study protocol was approved by Boston University Medical Center Institutional Review Board committee. HSC3 cells are highly aggressive SCC cancer cells with metastatic characteristics derived from human tongue and were generated by multiple successive passages of a tumor in mice^19^. LY2 cells are aggressive SCC cancer cells with metastatic characteristics and were derived from a BALB/c mouse keratinocyte tumor, and were kindly provided by Dr. Nadarajah Vigneswaran and Dr. Wolfgang Zacharias^20^. UMSCC2 cells are human OSCC cells obtained from Dr. Thomas Carey at the University of Michigan^21^ and SCC71 human oral cancer keratinocytes were obtained from Dr. Rheinwald at Harvard University^22^. SCC25 and CAL27 cells are human tongue SCC cells and were obtained from ATCC.

The growth medium used for gingival fibroblasts was Dulbecco’s modified Eagle’s medium (DMEM, high glucose 11965-092, Thermo Fisher Scientific), 10% fetal bovine serum (FBS, F0926, Sigma), 1% non-essential amino acid (NEAA, 11140050, Gibco), and 1% penicillin/streptomycin (15140122, Gibco)^12^. For serum depletion, HGF or cancer cells were washed two times with PBS and treated with serum-free DMEM containing 1% Penicillin/Streptomycin for 24 h. To produce conditioned media (CM), cancer cells were grown to 90% visual confluence. After washing twice with PBS, they were treated with serum-free DMEM containing 1% Penicillin/Streptomycin for 24 h and the media were collected. Additional reagents were recombinant human PDGF-BB (#100-14B) and PDGF-AB (100-00AB) were purchased from Peprotech. PDGF-CC (1687-CC-025) was purchased from R&D Systems. β-aminopropionitrile (BAPN) was purchased from Sigma/Aldrich.

### Cell proliferation assay

CyQUANT Cell Proliferation Assay kit (C7026, Life Technologies) measures the DNA content in cells in culture. Gingival fibroblasts were seeded at the density of 10,000 cells per well in a 24-well culture plate. After 6 h they were washed with PBS twice and treated with either HSC3 CM only, CM with PXS-S1C (1 µM), or CM with 5 μM PDGFR inhibitor Tyrphostin (also known as AG1296) for 24 h under a serum-free condition. The CM was aspirated and the cells were washed in PBS. Cells were then stained according to the CyQuant instructions. Cells were suspended and transferred to a well of a 96-well black microplate (Corning^®^ 96-Well Solid Polystyrene Microplate, CLS3915), fluorescence was measured with excitation at 480 nm and emission at 520 nm using Berthold Technologies TriStar LB 941 plate reeader.

### Real-time qPCR

HSC3 cells were washed with PBS and then treated with serum-free medium with or without PXS-S1C (1 µM) for 24 h. RNA was isolated using RNeasy Mini Kit according to the manufacturer’s instructions. cDNA was made by using High-Capacity cDNA Reverse Transcription Kit (4368814, Thermo Fisher Scientific). The cDNA concentration was measured by NanoDrop spectrophotometer and then subjected to qPCR using TaqMan Universal PCR Master Mix (4304437, Thermo Fisher Scientific) and TaqMan probes for LOX (Hs00942480_m1 Gene LOX), LOXL1 (Hs00935937_m1 Gene LOXL1), LOXL2 (Hs00158757_m1 Gene LOXL2), LOXL3 (Mm01184865_m1 Gene Loxl3), LOXL4 (Hs00260059_m1 Gene LOXL4), and 18S (Hs99999901_s1 18S Human probe) as a control.

### Measurement of protein levels and western blots

To measure protein concentration in CM, gingival fibroblasts, LY2, or HSC3 cells were washed with PBS two times and then serum-free medium (5 ml) was added to the cells and after 24 h were collected. The CM were concentrated from 25 to 1 ml using Amicon Ultra-15 Centrifugal filter devices (Z717185, Millipore Sigma) and protein concentrations measured with Nano Orange Protein Quantification kit (N6666, Invitrogen), and subjected to western blotting for LOXL2. Samples (20 µg) were analyzed.

To measure protein expression in cell layers, cells were washed with PBS and lysed with SDS–PAGE sample buffer. Protein level of the cell lysates was measured with the Nano Orange Protein Quantification kit. Then they were subjected to western blotting. Primary antibodies employed were anti-LOXL2 antibody (#GTX105085, GeneTex), rabbit mAb against phospho-PDGF Receptor β (Tyr751, #4549, Cell Signaling), rabbit mAb against phospho-PDGF Receptor β (Tyr771, #3173, Cell Signaling), rabbit mAb against phospho-PDGF Receptor α (Tyr849)/PDGF Receptor β (Tyr857, #3170, Cell Signaling), rabbit mAb against PDGF Receptor β (#3169, Cell Signaling), rabbit mAb against phospho-p44/42 MAPK (ERK 1/2, Thr202/Tyr204, #4377, Cell Signaling), anti-p44/42 MAPK antibody (ERK 1/2, #9102, Cell Signaling), anti-phospho-AKT antibody (Ser473, #9271, Cell Signaling), and rabbit anti-AKT mAb (#4691, Cell Signaling) Signals were visualized with HyGlo Quick spray (1001354, Denville Scientific) or SuperSignal™ West Femto Maximum Sensitivity Substrate (34095, Thermo Fisher Scientific) recorded with a G:BOX, Syngene, optimized so that the intensity of bands was not saturated. The membranes were stripped using Western Blot stripping buffer (21059, Thermo Fisher Scientific) and re-probed with antibodies against either β-tubulin (HRP Conjugate, #5346, Cell Signaling) or β-actin (#4970, Cell Signaling) as a loading control. Using ImageJ software, the band intensities were analyzed and normalized to β-actin or β-tubulin signals.

### Knockdown of PDGF A and PDGF B

A set of three PDGF A and two PDGF B MISSION small hairpin RNA (shRNA) lentiviral transduction particles (lentiviral-based shRNA vectors) were used to knock down PDGF A and PDGF B in HSC3 tumor cells. Hexadimethrine bromide (8 µg/ml) and PDGFB MISSION shRNA Lentiviral Transduction Particles Human (TRCN0000010411 and TRCN0000010412), PDGFA MISSION shRNA Lentiviral Transduction Particle Human (TRCN0000158353, TRCN0000157461, and TRCN0000156591) and MISSION^®^ pLKO.1-puro non-target shRNA control transduction particles were added to the cells at 2 MOI and incubated for 20 h at 37 °C in a humidified incubator with 5% CO_2_. The HSC3 cells were transduced at 70–80% visual confluence overnight. The medium was replaced with fresh medium and puromycin (final concentration of 2 µg/ml) was added the following day. Drug-resistant cells were expanded to become confluent.

### Sandwich ELISA for PDGF-AB

CM samples and standards were added to the 96-well ELISA plate (ThermoFisher, #EHPDGF AB) followed by incubation at 4 °C with gentle shaking overnight. The wells were washed and processed as described by the manufacturer. Absorbances were determined at wavelengths of 450 nm and at 550 nm and the concentration of PDGF-AB dimer in each sample determined using a standard curve generated at the same time.

### Pulldown assay

Gingival fibroblasts were serum starved and then treated with CM with or without PXS-S1C (1 µM) for 24 h. Fibroblast proteins were extracted and the carbonyl-containing proteins were bioyinylated with biotin hydrazide because the hydrazide (−NH−NH_2_) as a reactive group reacts with carbonyls to form hydrazine bond (R1R2C = NNH_2_) followed by sodium cyanoborohydride (NaBH_3_CN) reduction. Proteins were then subjected to pulldown assays using an avidin-coupled affinity resin (Neutravidin, Pierce Scientific). Blots of samples before and after purification were subjected to western blot and visualized with streptavidin-coupled HRP and anti-PDGFRβ antibody to assess for aldehydes and PDGFRβ-aldehydes in response to the CM treatment.

## Results

### LOX family expression in human OSCC

**Fig. 1:**
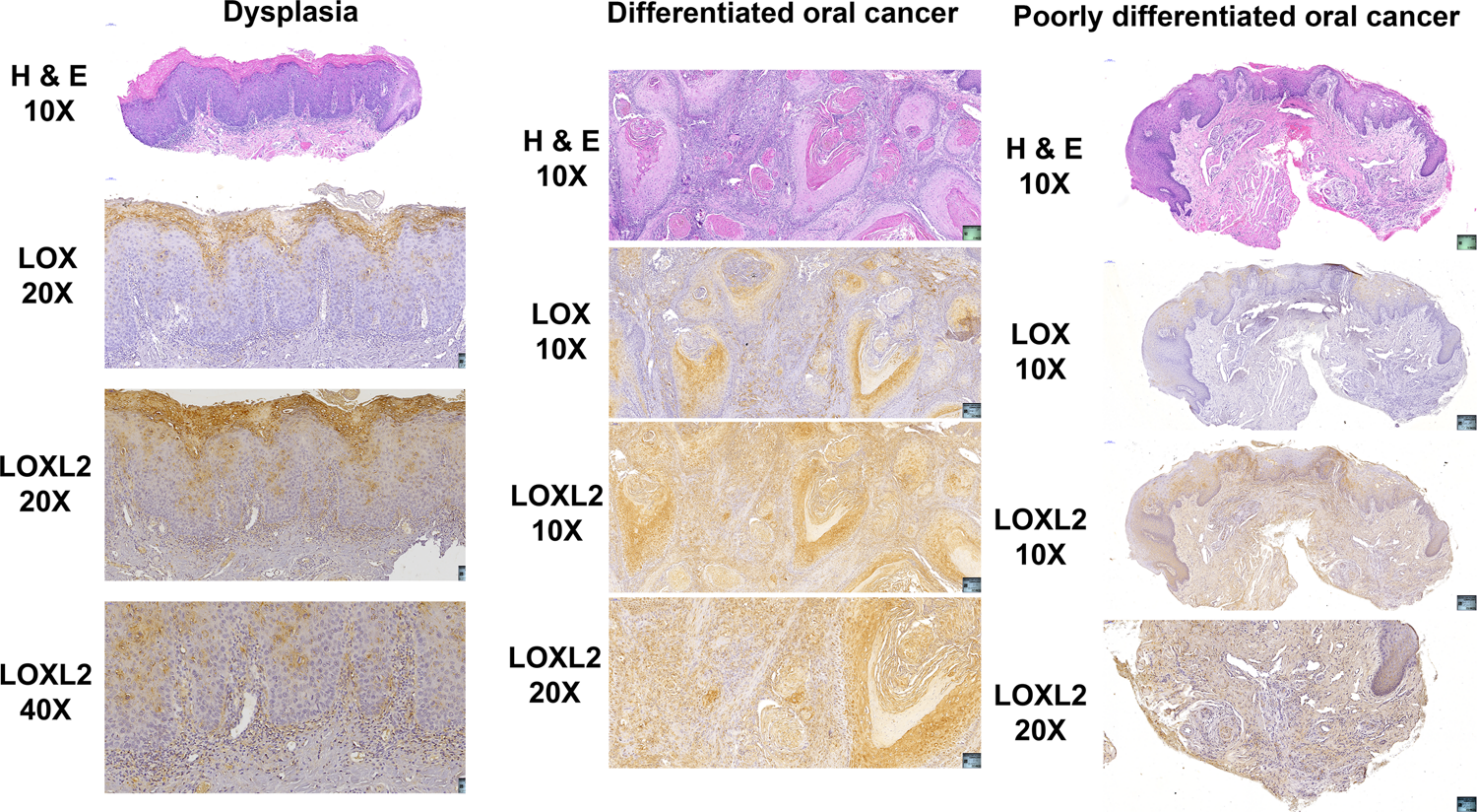
Histology and immunohistochemistry of human dysplasia and oral cancer biopsies for LOX and LOXL2. Biopsy samples were selected by the pathology service at Boston University Henry M. Goldman School of Dental Medicine and tissue sections were prepared and stained. Slides made from one selected subject from 3 to 5 subjects sampled in each category of dysplasia, differentiated oral cancer, and poorly differentiated oral cancer, respectively, are shown. Stained slides were imaged using an automated slide imager, and images were processed using Case Viewer software version 2.2 (Budapest, Hungary). Data indicate that LOXL2 was highly expressed in a variety of cancer cells and associated mesenchymal cells in human oral cancer, while LOX expression was more restricted

High levels of LOXL2 were previously associated with a poor prognosis of head and neck squamous cell carcinoma^23^. To independently determine if the other LOX paralogues were expressed in oral cancer, we investigated The Cancer Genome Atlas (TCGA) data set for OSCC of the tongue compared to non-affected tongue tissue from the same 334 subjects, focusing on the relative gene expression of all five LOX paralogues, LOX and LOXL1–LOXL4. Data in Table S1 demonstrate that in oral cancer tissues LOXL2 was by far the most significantly upregulated LOX paralogue (9.8-fold), while other paralogues were modestly upregulated (1.6–2.3-fold). To further characterize expression of LOXL2 and LOX in human tongue oral cancer, several biopsies obtained from the Boston University School of Dental Medicine Pathology diagnostic services laboratory were obtained under an approved IRB protocol. Figure 1 shows representative histopathology and IHC indicating that both LOX and LOXL2 were expressed in dysplasia, differentiated oral cancer and poorly differentiated oral cancer. In dysplasia, LOXL2 and LOX were highly expressed in the outer keratinized oral epithelium, with some staining observed extending into the *stratum spinosum*. LOXL2 was expressed by more cells than LOX consistent with TCGA data in Table S1. In differentiated oral cancer, LOX and LOXL2 were clearly expressed in tumor nests, and in associated elongated cells with a mesenchymal morphology. In poorly differentiated oral cancer, LOX staining appeared to be mostly restricted to the epithelium that exhibited light counterstaining consistent with keratinocyte edema. By contrast, LOXL2 staining occurred throughout the specimen in both tumor cells and in surrounding stroma. Taken together, TCGA and IHC studies of human squamous cell tongue cancer support that LOXL2 is expressed at high levels in poorly differentiated and well-differentiated oral cancer by tumor cells and mesenchymal cells, and further support the notion that LOXL2 likely contributes to the etiology of metastatic disease. High abnormal expression of LOX and LOXL2 was also observed in the epithelium of dysplastic tissue, suggesting a possible role in dysplasia that may ultimately be a factor in cancer development in combination with mutations and environmental influences. Due to the highest expression of LOXL2 in tongue oral cancer in the TCGA data, and the human histopathology and IHC in Fig. 1, we next focused on the study of expression and mechanisms by which secreted LOXL2 could promote oral cancer.

#### In vivo mouse studies

To define the effect of LOXL2 on oral cancer growth and metastasis, effects of the small molecule LOXL2 inhibitor PXS-S1C were investigated in both immunodeficient and immunocompetent mouse models. PXS-S1C is a novel small molecule active site-directed irreversible inhibitor whose structure is shown in Fig. S1A, and was provided to us by Pharmaxis Corporation, LLC, Australia. The specificity of the inhibitor is shown in detail in Fig. S1B from which Ic50s reported earlier were determined and compared to other LOX family enzymes, and other copper-dependent amine oxidases^12^. Irreversibility of LOXL2 inhibition by PXS-S1C was established in Fig. S1C in which the the Kitz–Wilson plot shows first-order inactivation kinetics consistent with PXS-S1C acting as an enzyme-activated irrevesible inhibitor^24^, with kinetic parameters shown in Fig. S1D.

### LOXL2 promotes tumor growth and oral cancer metastasis in immunodeficient mice

**Fig. 2:**
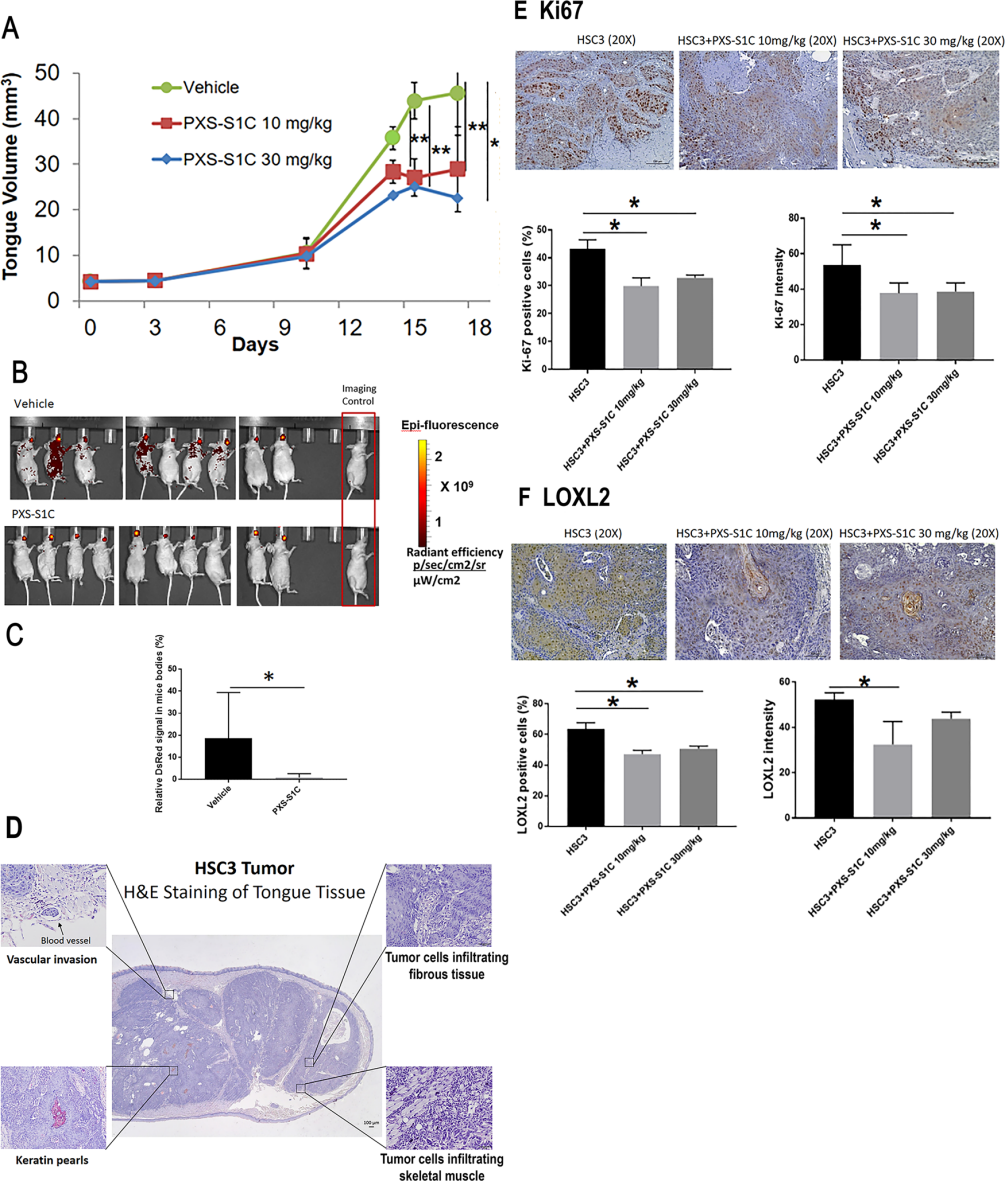
LOXL2 promotes human tongue orthotopic cancer growth and metastasis in mice. **a** PXS-S1C attenuates human tongue tumor growth in mice, and **b** and **c** PSX-S1C significantly decreases tumor cell spreading. Tumor volume of mouse tongues were measured every 3 days. Data are means ± SD. ANOVA, *p*: 0.0001, Tukey’s multiple comparisons test, **p < 0.001, ***p < 0.0001 indicate difference among the groups (*n* = 8 per group). **b** IVIS imaging for red fluorescent protein-labeled HSC3 cells 21 days after commencing injections of vehicle or PXS-S1C (30 mg/kg). The fluorescence signals were optimized for DsRed protein at excitation 570 nm and emission 620 nm. **c** Quantification of fluorescence signal area shows a significant difference between PXS-S1C-treated and non-treated groups. Data are mean ± SD. Student’s *t*-test, *p < 0.05 indicates difference between the groups. **d** Histology of HSC3 cell orthotopic tongue tumors. Hematoxylin and eosin staining a mouse tongue is shown 18 days after implantation. The photo is representative of histological features of HSC3 orthotopic tumors. The images were taken at ×4 and ×20 magnifications. Scale bar = 100 µm. PXS-S1C attenuates expression of (**e**) Ki67 and (**f**) LOXL2 in LY2 orthotopic tumors in immunodeficient mice. **e** Immunohistochemistry staining of tongue sections with anti-Ki-67 antibody shows that PXS-S1C reduced Ki-67 staining in orthotopic HSC3 tumors in mice. Scale bar = 100 µm. Data are mean ± SD. ANOVA, *p* < 0.01, Tukey’s multiple comparisons test, *p < 0.05 indicate difference among the groups (*n* = 8 per group). **f** Staining of tongue tissues with anti-LOXL2 antibody shows that PXS-S1C reduced LOXL2 staining in orthotopic HSC3 tumors in mice. Scale bar = 100 µm. Data are mean ± SD. ANOVA, *p* < 0.01, Tukey’s multiple comparisons test, *p < 0.05 indicate difference among the groups (*n* = 8 per group)

HSC3 cells are human aggressive tongue-derived OSCCs^19^, which were transduced to express RFP and were injected into the tongues of nude mice followed by injection of PXS-S1C (10 and 30 mg/kg) with a frequency of three times a week for 3 weeks by intraperitoneal injection. LOXL2 inhibition was found to attenuate tumor growth in tongues as determined by caliper measurements (Fig. 2a). Tracking cancer cells by IVIS after 3 weeks of injections of vehicle or PXS-S1C (30 mg/kg) demonstrated that inhibition of LOXL2 by PXS-S1C significantly decreased the distribution of cancer cells to other tissues (Fig. 2b, c).

#### Histopathologic analysis

H&E-stained sections of the primary HSC3 tumors showed features of differentiated infiltrating keratinizing squamous cell carcinoma. The tumors contained sheets and islands of atypical squamous epithelial cells infiltrating the connective tissue stroma and insinuating deep between skeletal muscle fibers. HSC3 tumor cells were infiltrative but demarcated given the lobular growth pattern and cellular cohesion. Keratin pearl formation and patchy dyskeratosis were appreciated. A mild predominantly acute inflammatory cell infiltrate occurred at the periphery of the tumor characterized by scattered polymorphonuclear leukocytes. Focal perivascular and perineural invasion were noted within tumors (Fig. 2d).

IHC staining of PXS-S1C-treated mice demonstrated reduced Ki-67 staining (proliferation marker) compared to no inhibitor-treated control mice (Fig. 2e) and lower expression of LOXL2 in tongue tumor samples in treated mice (Fig. 2f). The latter finding was unexpected, because PXS-S1C is a mechanism-based inhibitor of LOXL2 activity, and was not designed to directly target LOXL2 transcription.

### PXS-S1C inhibits oral cancer growth and metastasis in immunocompetent mice

**Fig. 3:**
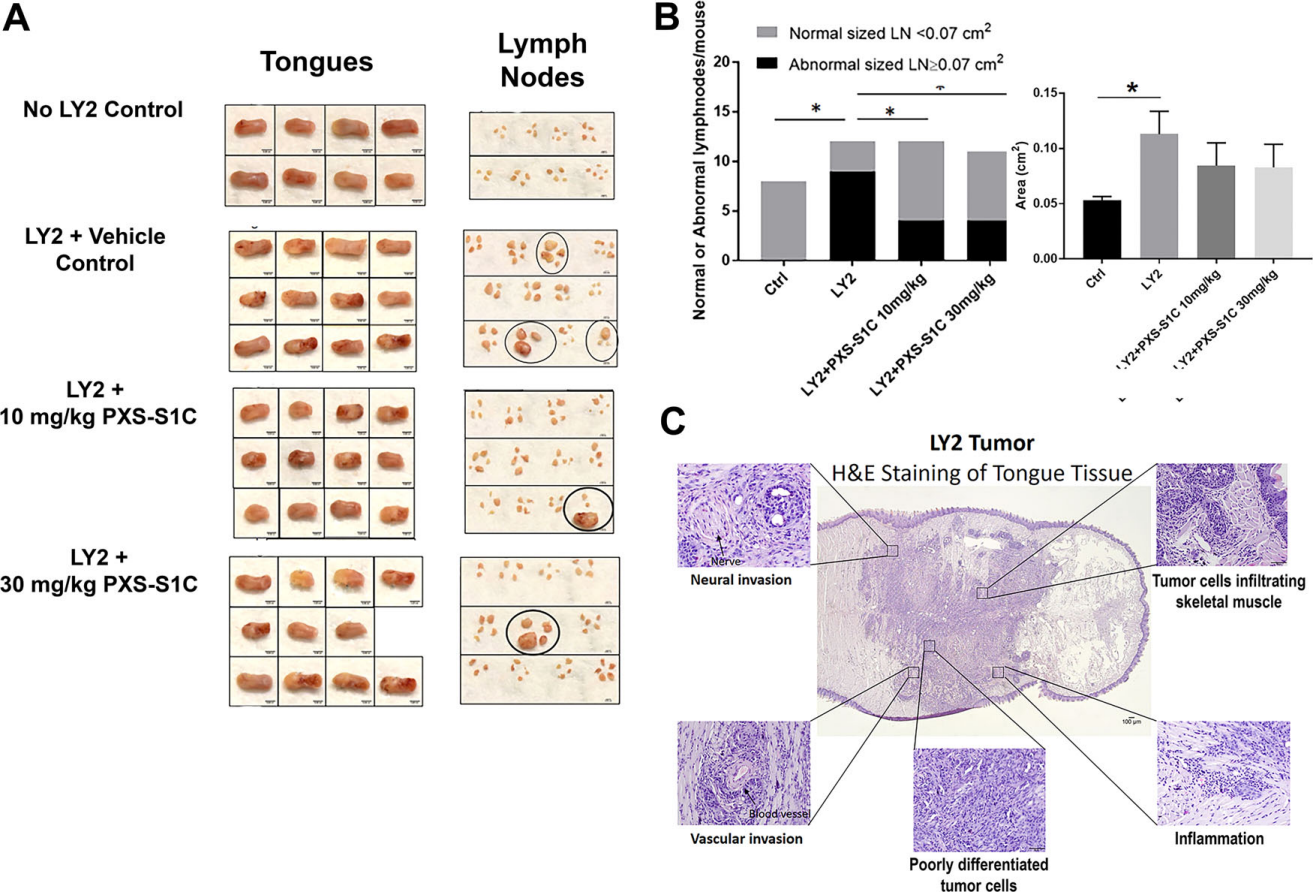
LOXL2 promotes syngeneic orthotopic tongue tumor growth and metastasis to cervical lymph nodes in immunocompetent mice. **a** Gross features of tongues and lymph nodes of the mice in all groups. Circles mark grossly oversized lymph nodes. **b** Number of the mice with abnormal size of lymph nodes (left panel) was reduced by the treatment with PXS-S1C. Number of the mice with normal versus abnormal size of lymph nodes. Normal sized LN in control mice = 0.053 ± 0.01 cm^2^, *p < 0.05. Chi-Square test (4 × 2 analysis) *p* = 0.008, Chi-Square test, *p* = 0.008, indicates difference in number of normal and abnormal sized LN among the groups. Average size of the lymph nodes in each group (right panel). ANOVA, *p* < 0.05, Tukey’s multiple comparison test *p < 0.05 indicates difference between the groups. Fisher’s exact test (2 × 2 analysis). Control vs. LY2, *p* = 0.001, mice injected with LY2 have larger LNs than controls. Fisher’s exact test (2 × 2 analysis) LY2 vs. both LY2 + PXS-S1C 10 and 30 mg/kg group ogether, *p* = 0.03, LOX inhibitor reduces the frequency of mice having enlarged LNs. Fisher’s exact test (2 × 2 analysis), LY2 + PXS-S1C 10 mg/kg vs. LOXL2 + 30 mg/kg, *p* > 0.05 There is no statistical difference between the two different doses of LOX inhibitor. **c** Histology of LY2 orthotopic tongue tumors. Hematoxylin and eosin staining of LY2 orthotopic tongue tumor. The images are representative of histological features of LY2 tumor. The images were taken at ×4 and ×20 objectives. Scale bar = 100 µm

The effect of PXS-S1C on cancer growth and metastasis in an immunocompetent mouse model was investigated by injection of LY2 to the tongue of syngeneic immunocompetent mice (Balb/c mice)^20^ followed by intraperitoneal injections of the LOXL2 inhibitor (10 and 30 mg/kg) three times a week as above, and mice were sacrificed after 6 weeks. Tongues exhibited lesions from all mice that had been injected with LY2 cells as expected (Fig. 3a). Interestingly, LOXL2 inhibitor treatment reduced the frequency of lymph node enlargement compared to the non-treatment group (Fig. 3a, b). The data suggest that LOXL2 enzyme activity significantly promotes oral cancer to cervical lymph node metastasis.

#### PXS-S1C lowers tumor LOXL2 levels and proliferating cancer cells

**Fig. 4:**
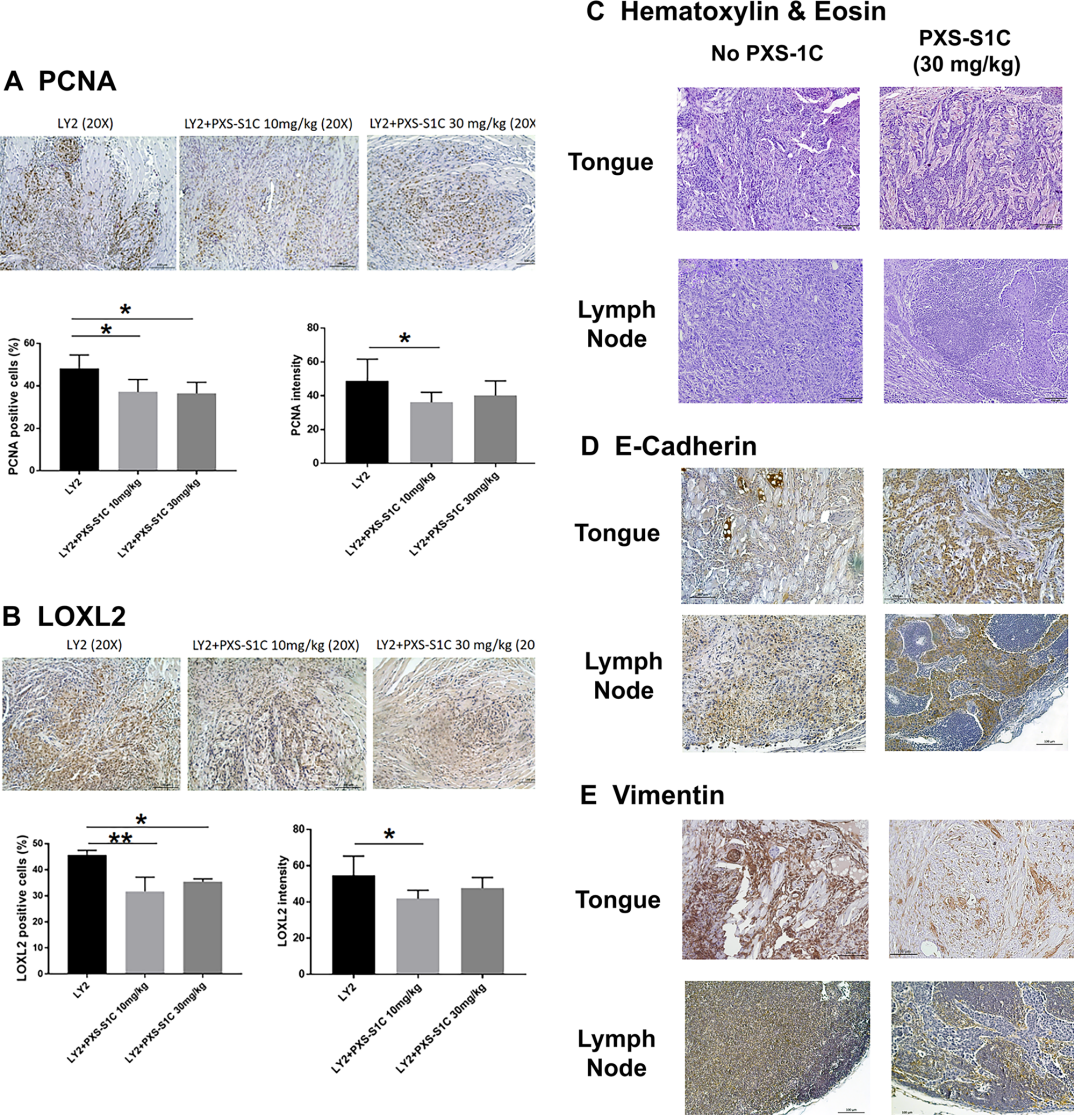
LOXL2 promotes proliferation and EMT of primary tongue and cervical lymph node metastases. PXS-S1C attenuates expression of (**a**) PCNA and (**b**) LOXL2 in LY2 orthotopic tongue tumors in immunocompetent mice after 6 weeks of treatment, scale bar = 100 µm. Data are mean ± SD. ANOVA, *p* < 0.05, Tukey’s multiple comparisons test, *p < 0.05 indicate difference among the groups (*n* = 12 per group). PSX-S1C treatment of LY2 tongue orthotopic tumors in mice alters tongue and cervical lymph node (**c**) tumor cell morphology, (**d**) E-cadherin expression and (**e**) vimentin expression. The images were taken at ×10 and ×20 magnifications. Scale bar = 100 µm. Images are from mice at the 6-week time point

H&E-stained sections of the primary LY2 tongue tumors showed histopathologic features of poorly to moderately differentiated infiltrating squamous cell carcinoma (Fig. 3c). The tumors revealed multifocal infiltrating islands of pleomorphic squamous epithelial cells. Atypical mitotic figures were readily identified. The tumor margins were poorly demarcated. The tumor cells were discohesive and haphazardly arranged and notable for single-cell invasion. LY2 tumors demonstrated significant perineural and perivascular invasion. The surrounding connective tissue stroma exhibited a patchy acute and chronic inflammatory infiltrate throughout (Fig. 3c). IHC staining of the tongue tissues indicate that treatment of mice with LOXL2 inhibitor PXS-S1C decreased levels of PCNA (Fig. 4a) and LOXL2 (Fig. 4b) in LY2 tongue tumors.

Inhibition of LOXL2 activity dramatically changed the morphological appearance of LY2 cancer cells in the tongue and lymph nodes. Treatment with PXS-S1C induced tumor cell changes consistent with transformation from a poorly differentiated to a well-differentiated morphology characterized by a more cohesive tumor cell arrangement at the border of the tumors in the tongues (Fig. 4c). In lymph nodes, tumor cells were arranged as sheets and islands of atypical squamous epithelial cells in the inhibitor-treated mice compared to a haphazard arrangement of tumor cells in untreated mice (Fig. 4c). These findings suggest that active LOXL2 promotes epithelial to mesenchymal transtition (EMT). To further assess for EMT, IHC staining of tongue tissues with E-cadherin and vimentin was carried out. IHC staining of tongue tissues shows that PXS-S1C treatment increased E-cadherin expression at the border of the LY2-derived tongue tumors and lymph node metastases (Fig. 4d). In contrast, PXS-S1C treatment reduced vimentin expression in the same tumors (Fig. 4e).

**Fig. 5:**
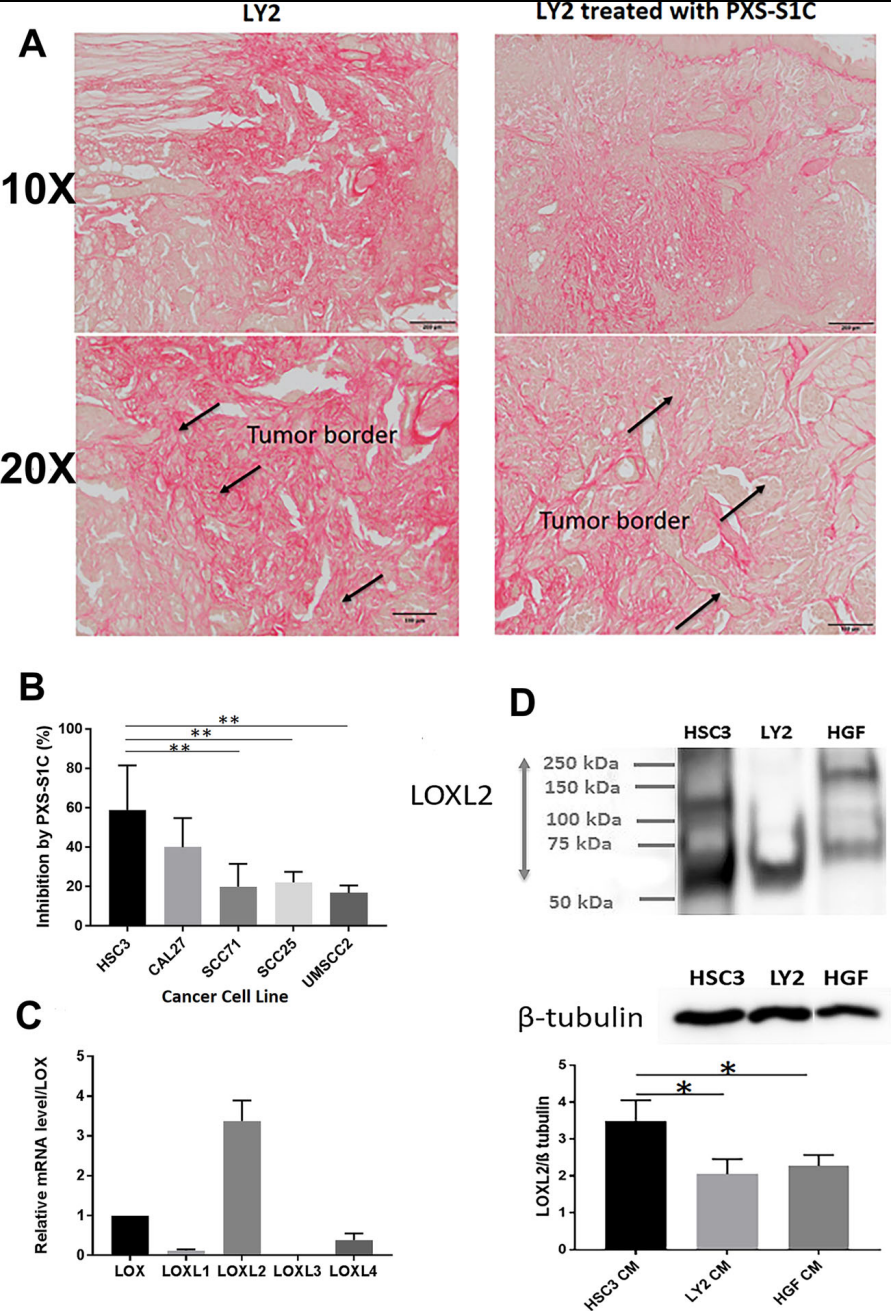
LOXL2 promotes collagen accumulation in syngeneic tongue oral cancer in mice, while human oral tumor cells-derived LOXL2 stimulates oral fibroblast proliferation in vitro. **a** Collagen accumulation in orthotopic tongue LY2 tumors by Sirius red staining of LY2 tumors in the tongue. A representative image is shown from one of 12 mice at the 6-week time point. Scale bar = 100 µm. Treatment with PXS-S1C appeared to reduce the amount of collagen, particularly at the apparent interfaces of tumor with surrounding non-tumor tissue. The images were taken at ×10 and ×20 magnifications. **b** Stimulation of human gingival fibroblast proliferation induced by CM of different oral cancer cell lines was inhibited by PXS-S1C treatment. Gingival fibroblasts were serum depleted for 24 h and then treated with cancer cell CM with and without PXS-S1C (1 µM) in serum-free conditions for 24 h, and DNA accumulation measured by CyQUANT assays: [(HSC3 CM)–(HSC3 + PXS-S1C)]/HSC3 CM × 100]. Data are means ± SD. Experiments were done with six replicate samples for each cell line. ANOVA, *p* < 0.0001, Tukey’s multiple comparison test. **p < 0.001 indicate differences among different groups. **c** LOXL2 is the most abundantly expressed paralogue by HSC3 cells. RNAs isolated from serum-depleted HSC3 cells was subjected to qPCR for all five lysyl oxidase paralogues using Taqman probes. Data are means ± SEM. This experiment was performed three times independently with triplicate samples. The RNA levels were normalized to 18S rRNA. ANOVA One way, *P* < 0.01 among all LOX family members. **d** LOXL2 protein is secreted at high levels by HSC3 cells. CM from human HSC3 cells, mouse LY2 cells and normal human gingival fibroblasts were collected under serum-free conditions, concentrated by 25-fold, and subjected to western blotting and visualized with anti-LOXL2 antibody. β-tubulin was used as a loading control. Data are means ± SEM. This experiment was done three times independently. ANOVA, *p* < 0.002, Tukey’s multiple comparison test *p < 0.05 indicate difference among the groups

Increased collagen accumulation is a hallmark of tumors prone to metastasize. In light of the function of LOXL2 in EMT and collagen synthesis, we investigated collagen accumulation in LY2 tumors. Measurement of Sirius red staining intensity in LY2 tumor and non-tumor areas showed that collagen accumulation increased in LY2 tumors in comparison with adjacent non-tumor regions (Fig. 5a). PXS-S1C treatment appeared to reduce collagen accumulation within and around LY2 tongue tumors (Fig. 5a).

### In vitro studies in human cells

#### Cancer cell-derived LOXL2 stimulates oral fibroblast proliferation and signaling

Fibroblasts are a major component of the cancer microenvironment that contributes to cancer progression and metastasis^25^. Due to high expression of LOXL2 in tumor cells observed above, and the fact that LOXL2 is principally a secreted enzyme, we next evaluated the hypothesis that LOXL2 secreted from tumor cells in some way directly targets surrounding fibroblasts. To assess for effects of tumor cell-derived LOXL2 on oral fibroblasts, the effect of human tumor cell-conditioned medium (CM) on the proliferative response of HGF in the presence or absence of the LOXL2 inhibitor PXS-S1C was next investigated.

#### Fibroblasts are targets of LOXL2

Cultured primary human oral fibroblasts were treated with non-CM, or cancer cell CM from the following oral cancer cell lines: HSC3, CAL27, SCC71, SCC25, and UMSCC2. PXS-S1C or vehicle was then added to CM to a final concentration of 1 µM. After 24 h of treatment, fibroblasts were subjected to CyQUANT DNA accumulation assays. Data indicate that PXS-S1C-attenuated human gingival fibroblast proliferative responses to all oral cancer cell CM tested. Proliferation of the HSC3 cell line was inhibited to the greatest degree by PXS-S1C (Fig. 5b). Mechanistic studies were next carried out with the HSC3 cell line due to its known aggressive character^19^ and the strong inhibition of proliferation observed.

#### LOXL2 mRNA and protein expression in oral cancer tumor cell lines

LOXL2 was found to be the highest LOX expressed by the HSC3 cell line (Fig. 5c). To assess the expression of LOXL2 protein in HSC3 and LY2 invasive oral cancer cell lines, and primary gingival fibroblasts, the cells were serum depleted for 24 h. Then the CM of cancer cells and gingival fibroblasts were collected and concentrated by ultrafiltration and subjected to western blotting for LOXL2. The data show that LOXL2 protein is secreted by HSC3 cells, and that HSC3 cells appear to express higher levels of LOXL2 than LY2 cells and HGF (Fig. 5d). LOXL2 was detected as multiple bands with different molecular weight because it undergoes posttranslational modifications including N-glycosylation, and proteolytic processing of its SRCR domains^26–28^.

#### Tumor cell secreted LOXL2 promotes fibroblast proliferation via PDGFR

To investigate the mechanism by which LOXL2 stimulates fibroblast proliferation in response to HSC3 CM, primary fibroblasts were serum depleted for 24 h and then treated with HSC3 CM for 5 or 10 min, and whole cell layer lysates were prepared. The effect on activation of signaling kinases was determined employing the receptor tyrosine kinase signaling array (RTK signaling array) indicated in the section “Methods”. Data show the activation of PDGFR, AKT, and ERK in human oral fibroblasts after treatment with CM, while other RTKs assayed were not activated under the conditions used (Fig. S2).

**Fig. 6:**
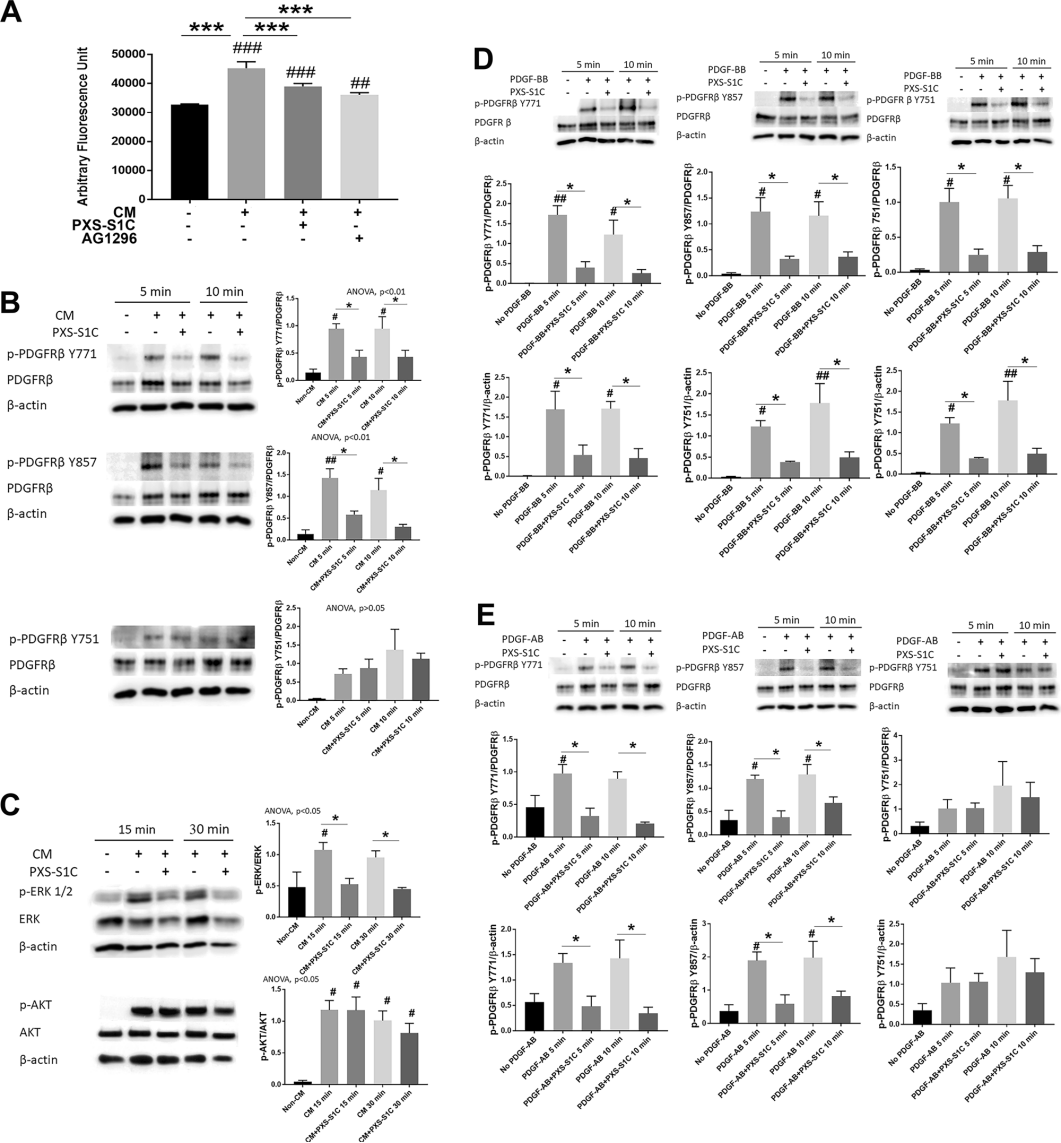
HSC3 oral tumor cell-derived LOXL2 stimulates oral fibroblast proliferation and cell signaling in collaboration with PDGF-AB. LOXL2 inhibitor PXS-S1C attenuates HSC3 CM-stimulated human oral fibroblast (**a**) proliferation, (**b**) phosphorylation of PDGFRβ at the Y771 and Y857 but not Y751 residues, and (**c**) ERK activation, but not AKT. **a** Human gingival fibroblast proliferation was reduced after 24-h treatment with PXS-S1C (1 µM) or AG 1296 (5 µM) in the HSC3 CM as determined by the CyQUANT assay. Data are means SEM. This experiment was done three times independently with six replicate samples. ANOVA, *p* < 0.0001, Dunnett’s multiple comparisons test, ***p < 0.0001 indicates significant difference between treated groups; while ^##^*p* < 0.001, ^###^*p* < 0.0001 indicate significant differences from non-CM group. **b** and **c** Gingival fibroblasts were serum depleted and then treated with non-CM, and CM with and without PXS- S1C (1 µM) and cell layer protein samples were subjected to western blot. Data are means ± SEM. The experiment was performed with three times independently with primary human gingival fibroblasts isolated from three different donors. Representative blots are shown. Data from all three experiments were subjected to quantitative analyses. Sidak’s multiple comparison test, *p < 0.05 indicates a significant difference from PXS-S1C treated group. Dunnett’s multiple comparison test, #*p* < 0.05, ^##^*p* < 0.001 indicate significant differences from Non-CM group. **d** PXS-S1C attenuates PDGF-BB stimulated phosphorylation of all three PDGFRβ phosphorylation sites Y771, Y857 and Y751, and AKT activation in oral fibroblasts. Gingival fibroblasts were serum depleted and then treated with no PDGF-BB, and PDGF-BB (10 ng/ml) with and without PXS-S1C (1 µM). The protein samples were subjected to western blot. Data are means ± SEM. The experiment was done with three times independently with primary human gingival fibroblasts isolated from three different donors. Representative blots are shown. Data from all three experiments were subjected to quantitative analyses. Sidak’s multiple comparison test, *p < 0.05, **p < 0.001, and ***p < 0.0001 indicate difference from PXS-S1C-treated group. Dunnett’s multiple comparison test, ^#^*p* < 0.05, ^##^*p* < 0.001, ^###^*p* < 0.0001 indicate difference from No PDGF group. **e** PDGF-AB mimics the effects of HSC3 cell CM on oral fibroblasts in phosphorylation of PDGFRβ. Gingival fibroblasts were serum starved and then treated with no PDGF-AB, and PDGF-AB (10 ng/ml) with and without PXS-S1C (1 µM). The protein samples were subjected to western blot. Data are means ± SEM. The experiment was done with three times independently with primary human gingival fibroblasts isolated from three different donors. Representative blots are shown. Data from all three experiments were subjected to quantitative analyses. Sidak’s multiple comparison test, *p < 0.05 indicates difference from PXS-S1C-treated group. Dunnett’s multiple comparison test, ^#^*p* < 0.05 indicates difference from No PDGF group

To investigate whether LOXL2 contributes to oral fibroblast stimulation by tumor cells via PDGFRs, oral fibroblasts were serum depleted for 24 h and then treated for 24 h with non-CM, CM, CM with PXS-S1C (1 µM) or CM with PDGFR inhibitor AG 1296 (5 µM). Following the treatment, the fibroblast proliferative response (DNA accumulation) was determined by CyQUANT assay. The data show that both PDGFR inhibitor and LOXL2 inhibitor treatments decreased the CM-stimulated proliferative response of oral fibroblasts (Fig. 6a). These data suggested that LOXL2 participates in tumor cell CM-induced stimulation of the proliferation of human oral fibroblasts by interacting with fibroblast PDGF signaling.

To assess whether inhibition of LOXL2 affects the PDGFR activation in response to CM, gingival fibroblasts were serum depleted and then treated for 5 or 10 min with no-CM, CM, or CM with LOXL2 inhibitor (1 µM PXS-S1C). Equal amounts of proteins were subjected to western blotting for phosphorylated (p-) PDGFRβ Y771, p-PDGFRβ Y857, p-PDGFRβ Y751, PDGFRβ, and β actin (loading control). PXS-S1C attenuated phosphorylation of PDGFRβ Y771 and βY857 in treated fibroblasts. However, PDGFRβ Y751 phosphorylation was unexpectedly not inhibited by PXS-S1C in response to HSC3 CM treatment of oral fibroblasts (Fig. 6b).

#### PXS-S1C attenuates fibroblast proliferation stimulated by cancer cell CM via ERK, but not AKT signaling

To further investigate the signaling pathway that mediates the decreased proliferation of HSC3 CM-treated oral fibroblasts treated with PXS-S1C, downstream signaling was assessed. Human serum-depleted oral fibroblasts were treated for 15 or 30 min with non-CM, CM, or CM with PXS-S1C (1 µM). The proteins were extracted and subjected to western blotting for activated AKT and ERK1/2. The blots showed that PXS-S1C surprisingly did not attenuate the activation AKT but did inhibit ERK1/2 signaling in fibroblasts treated with HSC3 CM (Fig. 6c). In summary, data indicate that LOXL2 activity present in HSC3-CM helps to promote activation of oral fibroblast PDGFR at Y771 and Y857 phosphorylation sites but not Y751 in PDGFR in response to CM treatment. Moreover, the LOXL2 activity helps to promote CM-stimulated fibroblast ERK1/2 phosphorylation, but not AKT phosphorylation.

#### PDGF-AB but not PDGF-BB mimics the effect of HSC3 cancer cell CM on oral fibroblasts

PDGF signaling is best known to activate AKT and not ERK1/2 in the context of cancer. We next considered the hypothesis that a novel PDGF ligand secreted by HSC3 cells may be responsible for the observed ERK1/2 activation in oral fibroblasts.

**Fig. 7:**
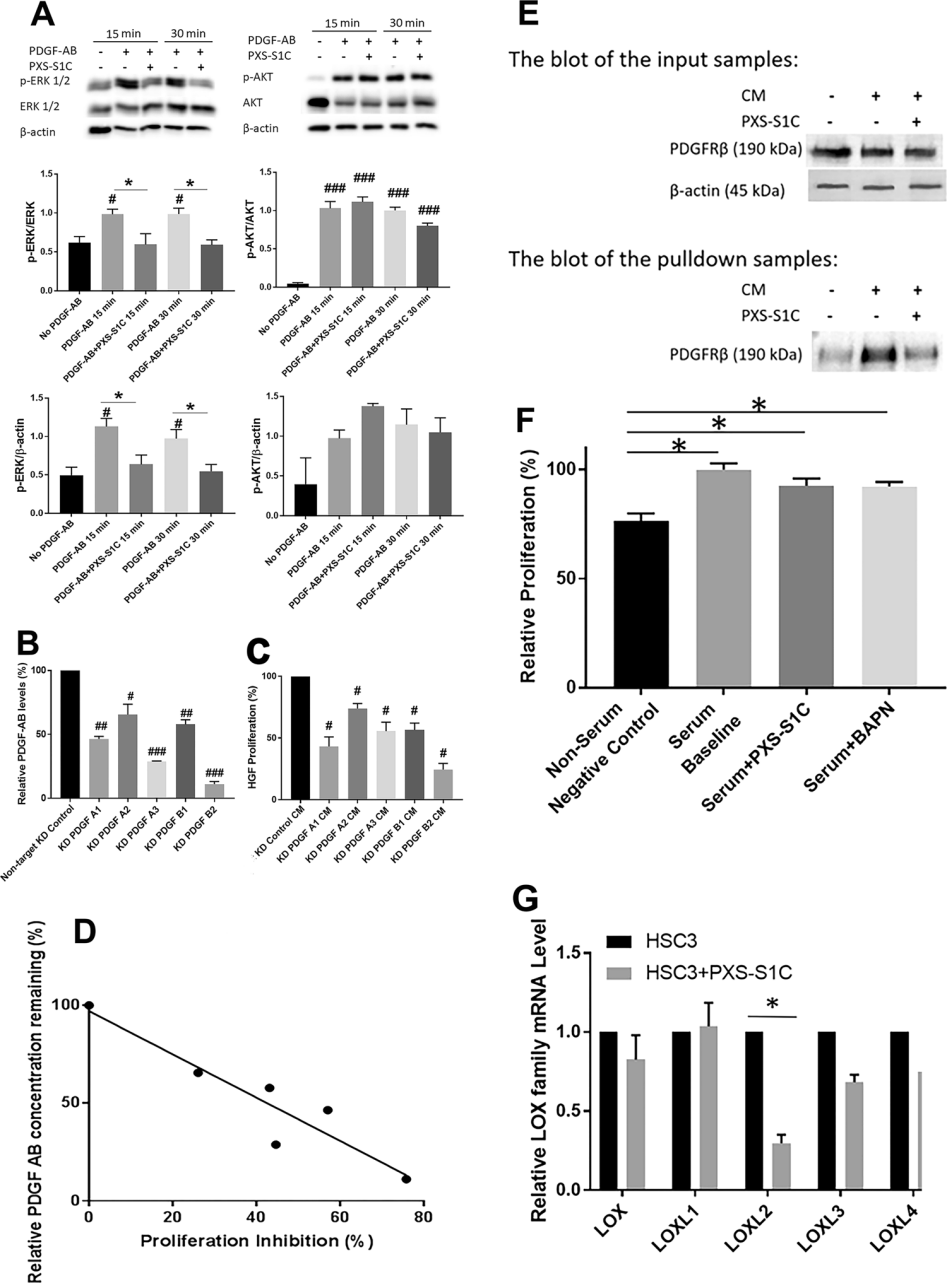
PDGF-AB, but not PDGF-BB, is secreted by HSC3 tumor cells, PDGFRβ is a substrate for LOXL2, and LOXL2 promotes its own synthesis in HSC3 cells. **a** PDGF-AB mimics the effects of HSC3 cancer cell CM on oral fibroblasts in phosphorylation of ERK1/2, and not AKT. Oral fibroblasts were serum depleted and then treated with no PDGF-AB, and PDGF-AB (10 ng/ml) with and without PXS-S1C (1 µM). The protein samples were subjected to western blot. Data are means ± SEM. The experiment was done with three times independently with primary human gingival fibroblasts isolated from three different donors. Representative blots are shown. Data from all three experiments were subjected to quantitative analyses. Sidak’s multiple comparison test, *p < 0.05 indicates difference from PXS-S1C-treated group. Dunnett’s multiple comparison test, ^#^*p* < 0.05 and ^###^*p* < 0.0001 indicate difference from No PDGF group. **b** PDGF-A and PDGF-B knockdown in HSC3 cells blocks HSC3 CM stimulation of oral fibroblast proliferation. The concentration of PDGF-AB ligand was decreased significantly in CM of knockdown HSC3 cells as compared with non-target control cell CM. PDGF-A and PDGF-B were, respectively, knocked down with independent shRNAs (A1, A2, or A3 for PDGF-A; and B1 and B2 for PDGF’B). The concentration of PDGF-AB ligand in knocked down HSC3 CM was measured using a PDGF-AB-specific ELISA kit. Data are mean ± SD. This experiment was done with triplicate samples. ANOVA, *p* < 0.0001, Dunnett’s multiple comparisons test ^#^*p* < 0.05, ^##^*p* < 0.001, ^###^*p* < 0.0001 indicate difference from Control HSC3 group. **c** The proliferation of oral fibroblasts treated with knocked down HSC3 medium for 24 h was assessed by CyQUANT assay. Data are mean ± SEM. This experiment was performed three times independently with primary human gingival fibroblasts isolated from three different donors. ANOVA, *p*: 0.0001, Dunnett’s multiple comparisons test: ^#^*p* < 0.05, indicate difference from HSC3 Control group. **d** The degree of PDGF-A or PDGF-B knockdown correlate linearly with decreased proliferative responses to HSC3 CM. The relationship between fibroblast proliferation inhibition and the relative level of PDGF-AB concentration was analyzed using linear regression. Correlation coefficient *r*: −0.93, *R* squared: 0.87, *p*-value: 0.006. Data indicate that PDGF-AB specifically is the ligand in HSC3 CM that stimulates oral fibroblast proliferation. **e** Carbonyl pull down assay for PDGFRβ in oral fibroblasts treated with HSC3 CM in the absence or presence of PXS-S1C. Human oral fibroblasts were treated with HSC3 CM in the absence or presence of 1 µM PXS-S1C followed by biotin hydrazide derivitization and affinity pulldown with a streptavidin affinity resin (Neutravidin). Input samples and proteins eluted by boiling in SDS–PAGE were subjected to Western blotting for PDGFRβ. Data are representative of two experiments with the same outcome from two different gingival fibroblast donors. **f** PXS-S1C and BAPN did not inhibit serum-stimulated proliferative response of HSC3 tumor cells. HSC3 cells were serum-depleted overnight and treated with PXS-S1C (1 µM) or BAPN (0.5 mM) in medium containing 2.5% serum for serum stimulation of a proliferative response. Data are means ± SEM. ANOVA, *p* < 0.0001, Tukey’s multiple comparisons *p < 0.05 indicates difference among the groups. **g** PXS-S1C decreased the expression of LOXL2 in HSC3 cells in vitro. Relative LOXL2 mRNA levels in HSC3 cell line with and without PXS-S1C after 24 h treatment was measured. Data are means ± SEM. This experiment was done three times independently with triplicate samples. ANOVA, *p*: 0.04, Sidak’s multiple comparisons test *p < 0.05 indicates difference from non-treated HSC3 group. The RNA levels were normalized to 18S rRNA

To evaluate the HSC3-secreted PDGF ligands that drive oral fibroblast PDGFR activation and with which LOXL2 may collaborate, the phosphorylation of PDGFRβ stimulated by different PDGF ligands with and without the LOXL2 inhibitor PXS-S1C was assessed. Serum-depleted human oral fibroblasts were treated with no PDGF, PDGF-BB, PDGF-CC, and PDGF-AB with vehicle or PXS-S1C (1 µM) and evaluated for activation of PDGFR, ERK 1/2, and AKT as above. Data indicate that PXS-S1C attenuated phosphorylation of all PDGFRβ phosphorylation sites including PDGFRβ Y751, and AKT in activating phosphorylations assayed in response to PDGF-BB (Fig. 6d), while PXS-S1C did not attenuate phosphorylation of PDGFRβ in response to PDGF-CC (Fig. S3). Interestingly, in response to HSC3 CM treatment of oral fibroblasts, PXS-S1C attenuated phosphorylation of PDGFRβ Y771 and βY857 but not PDGFRβ Y751 (Fig. 6e). PXS-S1C attenuated ERK1/2-related PDGF signaling, while AKT was not affected by PXS-S1C (Fig. 7a). Thus, data show that PDGF-AB, but not PDGF-BB or PDGF-CC, precisely mimics the effects of HSC3 CM on gingival fibroblasts.

#### PDGF-A or PDGF-B knockdown in HSC3 cells inhibits proliferation of oral fibroblasts induced by HSC3 CM

To confirm independently that PDGF-AB is the ligand secreted by HSC3 cells that stimulates oral fibroblast proliferation in collaboration with LOXL2 activity, shRNA lentiviral particles were used to knock down PDGF-A or PDGF-B in HSC3 cells. CM from knock-down cells were then assayed for PDGF-AB levels by ELISA, and the same media samples were assayed for the ability to stimulate proliferation of oral fibroblasts. The concentration of PDGF-AB ligand in knockdown and control HSC3 CM was next measured using a PDGF-AB ELISA which specifically recognizes the PDGF-AB dimer and not PDGF-AA or PDGF-BB. Data indicated that the concentration of PDGF-AB ligand was decreased significantly in the knocked-down HSC3 medium (Fig. 7b). Serum-depleted primary human oral fibroblasts were then treated with aliquots of the same CM of knock-down or control cells for 24 h and finally subjected to CyQUANT assay to assess proliferative responses to CM from knocked-down tumor cells. The result shows that fibroblast proliferation was significantly lower after PDGF ligand knockdowns in HSC3 cells in comparison with CM from HSC3 cells transduced with non-target shRNA control particles (HSC3 control) (Fig. 7c). To investigate whether the level of PDGF-A or PDGF-B knock down in HSC3 cells correlates with the proliferative response in fibroblasts treated with HSC3 CM, the relationship between the gingival fibroblast proliferation inhibition derived from Fig. 7b and the relative level of PDGF-AB concentration found in in Fig. 7c was analyzed by linear regression. The data indicate that the stronger knockdowns correlate well with lower proliferative responses to CMs (Fig. 7d). Taken together, data indicate that PDGF-AB is the major factor derived from HSC3 cells that drives oral fibroblast proliferation in collaboration with LOXL2. Interestingly, the DNA synthesis of knock-down HSC3 cells themselves showed no significant difference in cell proliferation compared to the no knock-down HSC3 control group (Fig. S4). Thus, PDGF-AB signaling did not affect proliferation of HSC3 cells, and PDGF-AB targets only fibroblasts in the microenvironment.

#### LOXL2 in CM oxidizes PDGFRβ in oral fibroblasts

We next considered the hypothesis that LOXL2 could optimize PDGF receptor signaling in response to PDGF-AB by oxidizing exposed lysine residues of the PDGFRβ receptor protein, possibly similar to that found for the LOX paralogue (not LOXL2) on smooth muscle cells and megakaryocytes^10,11^. We therefore developed an assay to identify LOXL2-dependent generation of aldehydes on PDGFRβ in oral fibroblasts treated with HSC3-CM. Oral fibroblasts were serum depleted and then treated with HSC3 CM with or without PXS-S1C (1 µM) for 24 h. Cell layer proteins were extracted and the aldehyde-containing proteins were affinity labeled with biotin hydrazide. Proteins were then subjected to a pulldown assay with an avidin-coupled affinity resin (Neutravidin), followed by SDS–PAGE and western blotting for PDGFRβ. Blots of samples before and after purification were subjected to western blot and visualized with anti-PDGFRβ antibody to assess for PDGFRβ-aldehydes in response to CM treatment. Western blots of the samples probed with anti-PDGFRβ antibody showed that LOXL2 inhibitor PXS-S1C decreased the oxidation of PDGFR compared to control samples lacking the LOXL2 inhibitor (Fig. 7e). We conclude that PDGFRβ is a substrate for LOXL2.

#### PXS-S1C effects on HSC3 cell growth and LOXL2 expression

We considered the notion that secreted LOXL2 could stimulate the proliferation of tumor cells in addition to neighboring stromal cells. To determine the effect of inhibition of LOXs and specifically LOXL2 on tumor cells, the serum-stimulated proliferative response of HSC3 cells in the presence or absence of PXS-S1C or BAPN, which inhibits all LOX paralogues including LOXL2, was investigated. HSC3 cells were serum-depleted overnight and treated with PXS-S1C (1 µM) or BAPN (0.5 mM) in medium containing 2.5% serum for serum stimulation of a proliferative response. After 24 h of treatment, the change in DNA accumulation was determined as a measure of the proliferative response by CyQUANT assay. The data indicate that neither PXS-S1C nor BAPN attenuated HSC3 cell growth in vitro (Fig. 7f). HSC3 cells grown in the presence of 2.5% serum in the presence or absence of PXS-S1C were next assessed for relative RNA levels of all five LOX paralogues. Interestingly, data indicate that PXS-S1C decreased the expression specifically of LOXL2 and no other paralogue in HSC3 cells in vitro (Fig. 7g). Data suggest that LOXL2 activity regulates its own expression in HSC3 tumors cells by a feed-forward autocrine pathway.

## Discussion

OSCC is one of the most common cancers in the world, with poor survival and a high recurrence rate^1,29^. Development of OSCC is typically evolves at a low frequency from hyperplasia and dysplasia to carcinoma in situ and finally to invasive metastatic OSCC. Oral cancer can also develop independent of this typical progression^30,31^. In either case, the development of OSCC is accompanied by changes in intercellular signaling between tumor cells and non-tumor cells, such as fibroblasts in the tumor microenvironment leading to dysregulation of gene expression and protein products facilitating cancer progression and metastasis^8,32,33^. Understanding of the molecular mechanisms underlying OSCC progression is essential to afford the opportunity to develop novel strategies to suppress tumor progression. The findings of previous studies have shown that LOXL2 is highly expressed in a variety of cancers in humans including head and neck squamous cell carcinoma, breast, colon, skin, and gastric cancer, and its high expression correlates with metastasis and poor prognosis^23,34–37^. Studies on overexpression of LOXL2 have not precisely indicated underlying mechanisms by which LOXL2 contributes to OSCC progression and metastasis. The direct targets of LOXL2 in cancer remain unclear. Therefore, we sought to investigate mechanisms by which LOXL2 induces progression and invasiveness in OSCC, and propose that LOXL2 is a potential target for OSCC therapy.

Immunocompromised and immunocompetent mouse models of oral cancer employed here both demonstrated significantly elevated LOXL2 levels, consistent with data in humans noted above and from Fig. 1 and the TCGA resource (Table S1). The application of a novel and highly selective small molecule inhibitor of LOXL2-attenuated tumor growth and metastatic spread to a significant degree in both mouse models. Although complete inhibition of cancer growth was not accomplished, the possibility is raised that LOXL2 inhibitors in combination with other therapeutic approaches including immunotherapy could result in potentially effective strategies to address oral cancer. The LY2 cells in the immunocompetent model appeared morphologically to feature characteristics of aggressive poorly differentiated cells, and appear to undergo what resembles a reversal of EMT in response to the LOXL2 inhibitor. This notion is supported by the increased levels of E-cadherin and lower levels of vimentin staining in both tongue tumors and lymph nodes observed in PXS-S1C-treated mice, and further supports the idea that LOXL2 significantly contributes to oral cancer development and that LOXL2 inhibitors may be of benefit in addressing oral cancer. LOXL2 promotion of EMT has been reported in several cancer models previously^14,35,38–43^, and the use of the novel selective LOXL2 inhibitor employed here resulting in apparent MET further supports the role of LOXL2 in cancer progression.

There is now a considerable body of literature indicating that elevated LOXL2 in particular is associated with a variety of cancers, including oral cancer. As reviewed previously by us and others^44–50^, proposed mechanisms range from enzymatic and non-enzymatic nuclear activities that promote EMT, to extracellular enzyme-mediated indirect or direct activation of FAK and proliferative responses in tumor cells. Molecular details of these relationships are still largely under investigation by a variety of research teams. Here, we entertained the notion that tumor cell-secreted LOXL2 may target non-tumor mesenchymal cells to stimulate proliferation. The resulting abundant fibrogenic cells, which produce collagens and LOXs and MMPs, would then ultimately modify the surrounding microenvironment by contributing to high collagen synthesis and cross-linking to promote an environment conducive to either cancer cell growth and/or metastasis. Our studies, led us step by step, to the finding that oral tumor cells secreted LOXL2 in combination with PDGF-AB, enhance ERK signaling and oral fibroblast proliferation. The mechanism of action of LOXL2 that enhances PDGF receptor sensitivity to PBGF-AB appears to consist of direct oxidation of lysine residues on PDGFRβ, similar to what occurs in normal vascular smooth muscle cells in response to the LOX paralogue^51^. The identity of the lysine residues in PDGFRβ that are oxidized remain to be determined and is under investigation.

### PDGF signaling as a driver of oral cancer

PDGF signaling has been reported to be a driver of EMT in normal development and in a variety of cancers^52–58^. In oral cancer in particular, elevated levels of PDGFRβ have been identified in associated stromal cells^59^, consistent with our hypothesis that PDGF ligands emanating from tumor cells have biological activity in the tumor microenvironment. Moreover, PDGF ligands drive metastatic oral cancer cell line migration mediated by PDGFRβ in tumor cells^58^. It was previously reported that optimization of PDGF signaling in vascular smooth muscle cells by LOX-dependent oxidation of PDGFRβ accompanied enhanced smooth muscle cell chemotaxis and migration. Note that LOX is not LOXL2. Thus, we show here for the first time that LOX and LOXL2 appear to share the ability to oxidize and optimize the function of PDGFRβ.

Our studies suggest that the outcome of PDGF signaling differs between oral tumor cells and oral fibroblasts. Stimulation of proliferation of fibroblasts, and stimulation of tumor cell EMT appear to be the respective outcomes. This conclusion is based on our extensive data shown above that LOXL2-stimulated proliferation of oral fibroblasts depends on LOXL2 activity and PDGF-AB activation of ERK. By contrast, in vivo modulation of tumor cell morphology, E-cadherin and vimentin levels, and the lack of in vitro inhibition of tumor cell proliferation by the LOXL2 inhibitor, point to a possible EMT function rather than proliferative stimulation of tumor cell growth. As noted, PDGFRβ in oral tumor cells has been shown to mediate migration, which can be a consequence of the EMT process^58^. An additional complexity is the apparent feed forward requirement of tumor cells for LOXL2 activity to maintain LOXL2 expression (Fig. 7g) that may be independent of PDGF signaling.

**Fig. 8:**
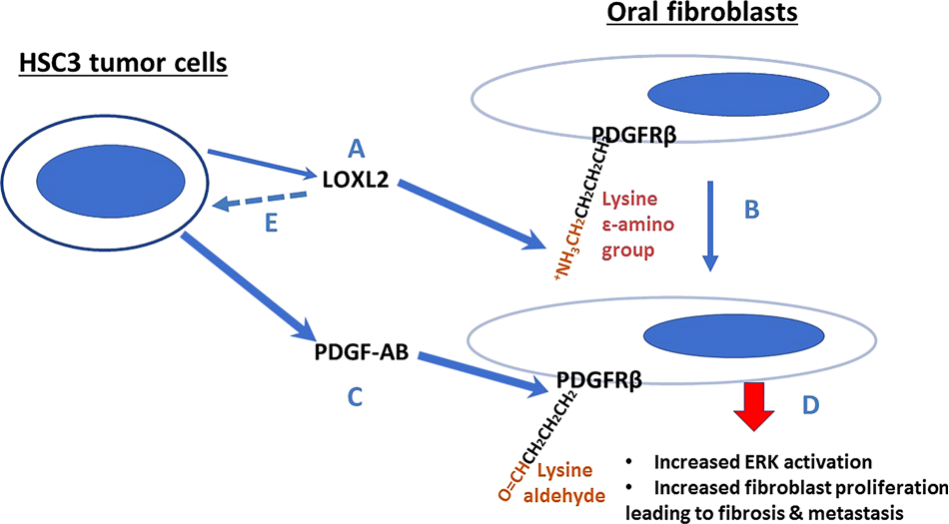
Summary of interactions between oral tumor cells and fibroblasts that contribute to oral cancer development. LOXL2 secreted by tumor cells (**a**) oxidizes lysine residues on PDGFRβ in proximal fibroblasts (**b**), in addition, to its classical role in collagen maturation (not shown). PDGF-AB secreted by tumor cells is consequently able to more efficiently stimulate PDGF signaling (**c**), resulting in increased ERK1/2 activation and cell proliferation (**d**). LOXL2 production by tumor cells is required for maintaining LOXL2 synthesis is a feed-forward pathway (**e**), whose mechanism remains to be determined

Figure 8 provides a summary of the new understanding of an extracellular role for LOXL2 in promoting oral cancer. In summary, we show that tumor cell LOXL2 targets proximal mesenchymal fibrogenic cells by a novel microenvironment tumor-promoting mechanism, while small molecule LOXL2 inhibition can improve oral cancer outcomes. Moreover, this study provides the first molecular mechanism for enzymatically active LOXL2 in the promotion of cancer via its modification of a non-collagenous substrate in the context of paracrine signaling between tumor cells and resident fibroblasts. The successful use of a selective LOXL2 pharmacologic inhibitor to address oral cancer in two in vivo mouse models provides pre-clinical evidence that supports the notion that similar inhibitors may have therapeutic potential against oral cancer.

## Supplementary information

Supplementary Figures
