# Supplementary material for: Mechanism for oral tumor cell lysyl oxidase like-2 in cancer development: synergy with PDGF-AB
Source: Oncogenesis. 2019 May 13;8(5):34. doi: 10.1038/s41389-019-0144-0 (PMC6513832; doi:10.1038/s41389-019-0144-0)
Supplement: Supplementary file 1 — Supplementary Figures [file 41389_2019_144_MOESM1_ESM.pdf]

Supplementary Materials

Materials and Methods

Selectivity of PXS-S1C. The concentration of PXS-S1C vs percent inhibition of all five lysyl oxidases was determined as described (1). The sources of enzymes employed was described previously (2). Time dependent irreversible inactivation of LOXL2 to generate a Kitz-Wilson plot was performed by preincubation with recombinant LOXL2 at 37°C for different intervals, followed by dilution to non-inhibitory PXS-S1C concentration and immediate enzyme assay using diaminobutane as substrate (3). Kinetic parameters were calculated from transformed data as described (4).

Analysis of human TCGA. The human oral cancer TCGA data set (5) was employed to assess for LOX and LOXL1 – LOXL4 gene expressions in base of the tongue tumors vs non-tumor adjacent tissues to independently assess for elevated expressions in oral cancer as described previously (6). Differential expression testing was performed with respect to tumor status (N = 334) using the limma R package (performed in log2 space after adding a pseudocount of 1 to the normalized expression matrix) as described previously.

Signaling array. PathScan® RTK Signaling Antibody Array Kit (Chemiluminescent Readout, Cell Signaling, #7982) is a screening tool used to detect activated receptor tyrosine kinases (RTKs). RTKs are captured by antibodies spotted on the membrane. The cells were washed with PBS and then scraped using cell lysis buffer (#9803, Cell Signaling) containing 20 mM Tris-HCl (pH 7.5), 2.5 mM sodium pyrophosphate, 1 mM β-glycerophosphate, 150 mM NaCl, 1 mM EGTA, 1 mM Na_2EDTA, 1 mM Na_3VO_4, 1% Triton X-100, and 1 µg/ml leupeptin. Then the samples were centrifuged at maximum speed for 10 minutes at 4°C and the supernatant were transferred into a new tube and diluted to 1mg/ml in diluent buffer.

Array blocking buffer was added to each well of the assembled array for 15 minutes, and the array was incubated with diluted lysate for 2 hours at room temperature. The array was washed

with array wash buffer and incubated with a detection antibody cocktail. After incubating with HRP-linked Streptavidin for 30 minutes, LumiGLO and Peroxide reagent was used to visualize the signal by chemiluminescence.

Supplementary Data:

Table S1.

TCGA analyses of lysyl oxidase paralogues expression in tongue tumors compared to adjacent non-tumor tissue.

Gene	Fold change mRNA vs adjacent normal tissue	Adjusted p Value
LOXL2	8.9	1.32E-28
LOXL3	2.3	2.18E-07
LOX	2.3	1.77E-04
LOXL1	1.6	0.022037
LOXL4	1.7	0.087474

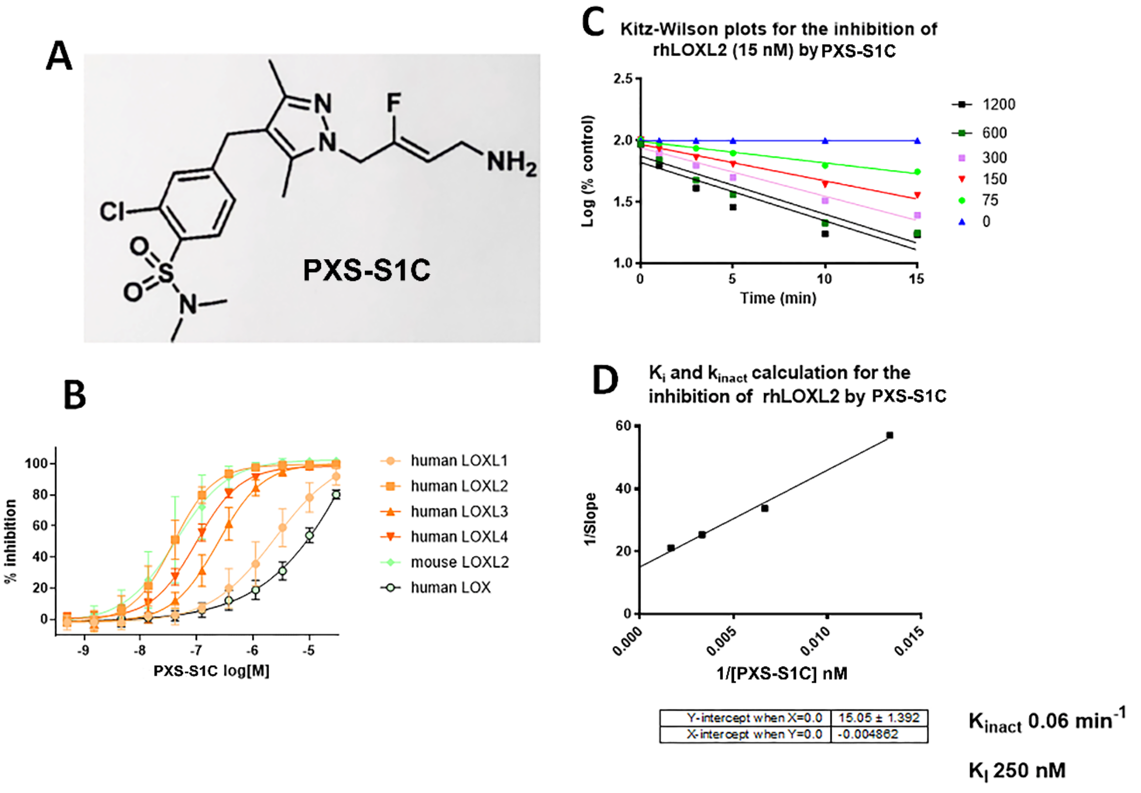

Figure S1. **(A)** Chemical structure of PXS-S1C. **(B)** Concentration curve for inhibition of all five lysyl oxidase enzymes. **(C)** Irreversible inactivation of recombinant LOXL2 by PXS-S1C (Kitz-Wilson plot) and **(D)** determination of kinetics of inactivation.

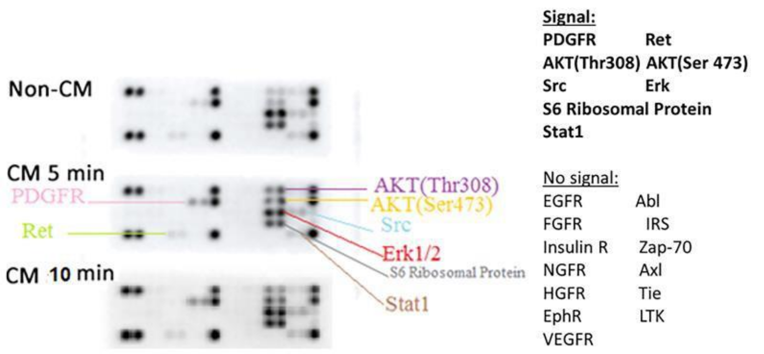

Figure S2. Tumor cell CM-stimulated tyrosine kinases inhibited by PXS-S1C. Serum depleted oral fibroblasts were treated with HSC3 cell CM and the protein sample was extracted and subjected to RTK signaling array. Phosphorylated PDGFR was detected after exposure of oral fibroblasts to CM of oral cancer.

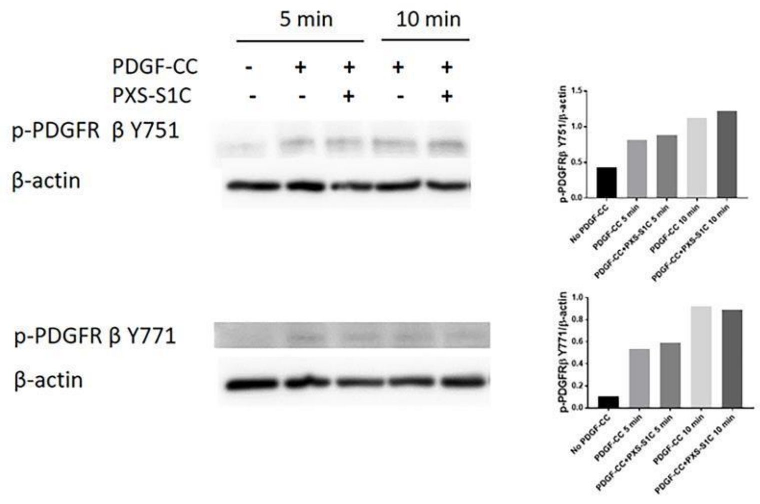

Figure S3. PXS-S1C does not attenuate phosphorylation of PDGFRβ in response to PDGF-CC treatment of oral fibroblasts. The gingival fibroblasts were serum starved and then treated with no PDGF-CC, and PDGF-CC (10 ng/ml) with and without PXS-S1C (1 μM). The protein samples were subjected to western blot. The experiment was done one time with primary human gingival fibroblasts isolated.

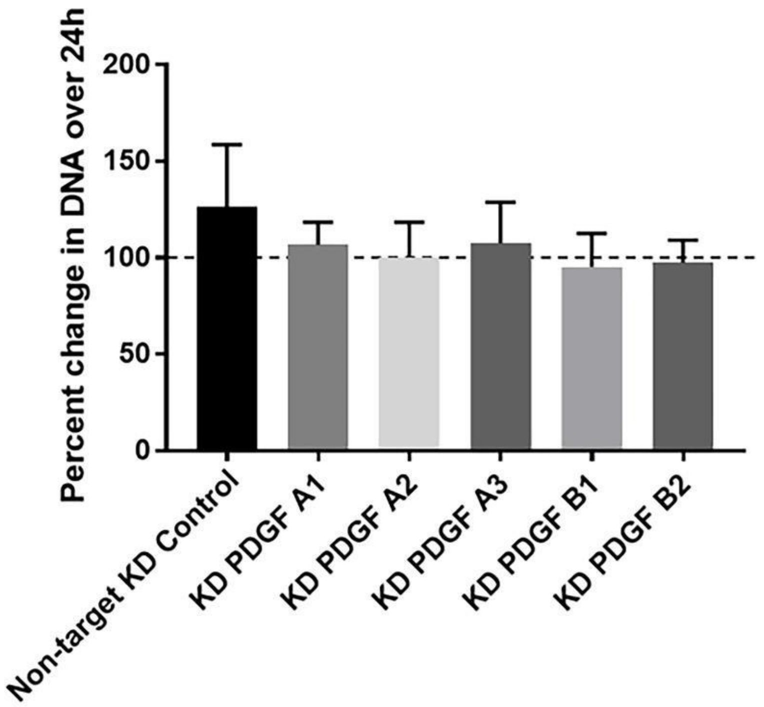

Figure S4. There is no significant change in cell proliferation in knock down cells.
Gingival fibroblasts were transduced with PDGF A (A1, A2, or A3), PDGF B (B1 or B2), or non-target (control). The resistant cells were isolated after puromycin treatment. The DNA synthesis of knock down cells was determined by CyQUANT assay and the percent changes inDNA synthesis were measured over 24 hours in serum free media. Data are means +/- SEM. This experiment was done 3 times independently with triplicate samples. ANOVA, p = 0.2.

Supplementary References

1. Schilter H, Findlay AD, Perryman L, Yow TT, Moses J, Zahoor A, et al. The lysyl oxidase like 2/3 enzymatic inhibitor, PXS-5153A, reduces crosslinks and ameliorates fibrosis. Journal of cellular and molecular medicine. 2019;23(3):1759-70.
2. Saxena D, Mahjour F, Findlay AD, Mously EA, Kantarci A, Trackman PC. Multiple Functions of Lysyl Oxidase Like-2 in Oral Fibroproliferative Processes. Journal of dental research. 2018:22034518775971.
3. Palamakumbura AH, Trackman PC. A fluorometric assay for detection of lysyl oxidase enzyme activity in biological samples. Anal Biochem. 2002;300(2):245-51.
4. Kitz R, Wilson IB. Esters of methanesulfonic acid as irreversible inhibitors of acetylcholinesterase. The Journal of biological chemistry. 1962;237:3245-9.
5. Comprehensive genomic characterization of head and neck squamous cell carcinomas. Nature. 2015;517(7536):576-82.
6. Hiemer SE, Zhang L, Kartha VK, Packer TS, Almershed M, Noonan V, et al. A YAP/TAZ-Regulated Molecular Signature Is Associated with Oral Squamous Cell Carcinoma. Molecular cancer research : MCR. 2015;13(6):957-68.
